# Methyltransferase like 3 promotes thyroid folliculogenesis *via* coordinating cell differentiation and polarization

**DOI:** 10.1515/jtim-2026-0005

**Published:** 2026-02-13

**Authors:** Ruoyu Jiang, Qibo Zhu, Zhenlei Zhang, Xiao He, Yifan Liu, Ronglin Kan, Xianghui He, Haixia Guan

**Affiliations:** Department of General Surgery, Tianjin Medical University General Hospital, Tianjin Medical University, Tianjin, China; Department of Endocrinology, Guangdong Provincial People's Hospital, Guangdong Academy of Medical Sciences, Southern Medical University, Guangzhou, Guangdong Province, China; Department of Thyroid and Neck Oncology, National Clinical Research Center for Cancer; Key Laboratory of Cancer Prevention and Therapy, Tianjin's Clinical Research Center for Cancer, Key Laboratory of Immune Microenvironment and Disease, Tianjin, China

**Keywords:** METTL3, congenital hypothyroidism, cell differentiation, cell polarity

## Abstract

**Background and Objectives:**

Congenital hypothyroidism (CH) is the most common neonatal endocrine disorder with largely elusive underlying mechanisms, although thyroid dysformation has been deemed as the most frequent cause. Methyltransferase like 3 (METTL3) serves as a pivotal writer for N^6^-methyladenosine (N6-methyladenosine, m^6^A) required for various organ development, but little is known about the significance of METTL3 and m^6^A modification in thyroid formation, in CH either. In this study, we aimed to clarify the new regulatory role of METTL3 in the occurrence and development of CH, and to provide new theoretical support and treatment ideas for the clinical treatment of CH.

**Methods:**

Thyrocyte-specific *Mettl3* knockout mouse model was constructed and subjected to morphological and functional analyses. Representative differentiation, polarization, and hormone synthesis factors were studied *via* immunohistochemistry, immunofluorescence staining, RT-qPCR, and thyroid hormone in serum were quantified. *In vitro*, function of *Mettl3* and molecular mechanisms were further investigated through thyrocyte cells from different species *via* lentivirus mediated silencing and rescue experiments.

**Results:**

Thyrocyte specific removal of *Mettl3* caused a typical CH phenotype, with reduced thyroid hormones and body weight. Histologically, the thyroid follicle of *Mettl3* deficient mice appeared as abnormally fused and enlarged structure, with significantly disturbed polarity and patterning. Mechanistically, Pax8 expression was reduced upon METTL3 loss due to damaged m^6^A modification, which resulted in compromised thyroid epithelial cell polarization, differentiation and hormone synthesis.

**Conclusions:**

*Mettl3* functions as a key player of thyroid folliculogenesis and hormone secretion by coordinating thyrocyte polarization and differentiation progression, and its deficiency may lead to congenital hypothyroidism.

## Introduction

Congenital hypothyroidism (CH) is the most common neonatal endocrine disorder, with thyroid hormone deficiency as the main clinical characteristics. CH affects about 1 in 2000 to 4000 newborns and mostly at a permanent way with lifelong.^[1]^ With delayed or inappropriate treatment, CH could lead to intellectual disability and growth retardation.^[2]^ Although the exact cause of CH remains elusive, developmental abnormalities of the thyroid gland, *i.e*., thyroid dysgenesis, as well as abnormal thyroid hormone synthesis accounts for the majority of the known cases.^[3, 4, 5]^ Genetic mice models have shown that the critical transcription factors, such as FOXE1 (TTF–2), NKX2–1 (TTF1), PAX8, NKX2–5, DUOX2, as well as related regulators like GLIS3 are essential for thyroid development, thus their mutations or functional loss could cause CH.^[6, 7, 8, 9, 10]^ Similarly, mutations in SLC5A5, thyroglobulin (TG), DUOX2, DUOXA2, SLC6A4, SLC26A7 and DEHAL1, which have been implicated in thyroid dyshormonogenesis, may also result in CH development.^[6,11, 12, 13, 14]^ However, clinical evidence shows that less than half of identified CH patients present mutations within these genes. Thus, other genes and/ or mechanisms could be involved in the pathogenesis of CH.^[9,15,16]^

Methyltransferase like 3 (METTL3) mediated m^6^A modification has been shown to play critical roles in various organ development and tumorigenesis.^[17]^ METTL3 is reported to regulate fetal growth,^[18]^ and become involved in the development of neurons, liver, brown adipose tissue, cardiomyocyte, and invariant natural killer T cell.^[19, 20, 21, 22, 23]^ METTL3 is also a widely investigated tumor suppressor in the tumorigenesis and immune microenvironment development of thyroid cancer.^[24, 25, 26]^ However, the role of METTL3 in the development of thyroid gland remains unclear.

In this study, we constructed an *Mettl3* thyroid-specific conditional knockout mouse model to investigate the role of METTL3 in thyroid development and CH, as well as to explore its potential underlying mechanism.

## Materials and methods

### Mice

Animals used in this study were maintained as specific-pathogen free (SPF) mice. Thyroid peroxidase (TPO)-Cre mice were a gift from Professor Shioko Kimura of NIH, USA. *Mettl3*^flox/flox^ mice were a gift from Professor Xiangqian Zheng of Tianjin Medical University Cancer Institute and Hospital, China.

### Cell lines

HEK293T (ACS-4500) was purchased from the American Type Culture Collection (ATCC), and cultured in Dulbecco’s modified Eagle’s medium (DMEM) supplemented with 10% fetal bovine serum (FBS) and 1% penicillin/streptomycin. The Fischer rat thyroid follicular cell line (FRTL-5) was purchased from Shanghai Yubo Biotechnology Co., LTD, and cultured in Leibovitz’s L-15 medium supplemented with 10% FBS and 1% penicillin/streptomycin. Human thyroid follicular epithelial cells Nthy-ori 3-1(Nthy), acquired from Tianjin Medical University Cancer Institute and Hospital, and cultured in Roswell Park Memorial Institute (RPMI) 1640 supplemented with 10% FBS and 1% penicillin/streptomycin. All the cells were maintained in a humidified incubator equilibrated with 5% CO_2_ at 37 °C.

### Plasmids

The plasmids expressing Flag-PAX8 were cloned in lentiviral vector pLV-EF1α-MCS-IRES-Bsd. Recombinant lentiviruses expressing different shRNAs were obtained by cloning designed shRNA into pLKO.1-puro. Related shRNA sequences are listed in Supplementary Table S1.

### Single cell RNA sequencing (scRNA-seq) data analysis

scRNA-seq matrix data were downloaded from the GEO database with search number GSE183963 (Mouse single-cell). Data normalization, dimensional reduction, batch effect removal, clustering, and visualization were performed with the Seurat 4 package. The Harmony method was used for batch effects correction to confirm clustering consistency. Seurat’s DimPlot function was used to generate the tSNE. We identified endothelial cells (Flt1, Kdr, Podxl), fibroblasts (Dcn, Col1a1, Col1a2), macrophages (C1qa, C1qb, Cd68), thyroid follicular cells (Epcam, Chchd10, Tg), which represent all major cell types in thyroid organoids.

### RT-qPCR

A quantitative real-time RT-PCR method was used to measure the amount of RNA. Briefly, total RNA was extracted using TRIzol (Invitrogen). Reverse transcription of RNA was performed using the RevertAid First Strand cDNA Synthesis Kit (Thermo Fisher Scientific). Relative quantitation was determined using the LightCycler 480 real-time PCR system (Roche) and then calculated by means of the comparative Ct method (2^-ΔΔCt^) with the expression of GAPDH or β-Actin as controls. Each sample was examined at least in triplicate. Primer sequences are listed in Supplementary Table S2.

### Western blot

Cells were lysed on ice using RIPA lysis buffer (Solarbio, Beijing, China) with freshly added proteinase inhibitors cocktail (Roche). The protein extracts were clarified and quantified by Pierce BCA Protein Assay (Thermo Scientific). Western blot was performed as previously described.^[27]^ The following antibodies were used: Anti-METTL3 (#ab195352, Abcam), Anti-PAX8 (#ab53490, Abcam), Anti-TTF-1 (#ab76013, Abcam), Anti-TPO (#ab278525, Abcam), Anti-NIS (#MA5–12308, Thermo), Anti-β-Actin (#3700, CST), Anti-Vinculin (#A14193, ABclonal).

### Sphere culture and assay

The cells were cultured in a stem cell culture medium in an ultralow attachment 24-well plate with a density of 100, 000 cells per well. The cell cultures were supplemented with 10 μg/L EGF, 100 IU/mL penicillin, 100 g/mL streptomycin, and B-27 Supplement (50 x) (Life Technologies, without serum). Photographs were taken after 48 h of incubation in Wells.

### Immunohistochemistry and immunofluorescence analysis

Tissue specimens from mouse models were fixed in 4% PFA and embedded in paraffin. Immunohistochemistry and immunofluorescence were performed following a standard procedure.^[28]^ Sections of 5 μm were stained with hematoxylin and eosin (H & E) and incubated with primary antibodies including: Anti-METTL3 (#ab195352, Abcam), Anti-PAX8 (#ab53490, Abcam), Anti-TTF-1 (#ab76013, Abcam), Anti-TPO (#ab278525, Abcam), Anti-TG (#ab156008, Abcam), Anti-β-Catenin (#ab32572, Abcam), Anti-GM130 (#ab52649, Abcam), Anti-EZRIN (#ab40839, Abcam), Anti-ZO-1 (#ab221547, Abcam).

### Scratch-wound assay and polarity index calculation

To assess functional collective cell behavior properties (*i.e*., polarity and migration), as well as morphological features of *in vitro* cultured FRTL-5 cell, we used the scratch-wound assay. The wound was created by scratching the surface of a microscopy glass slide containing a monolayer of adherent FRTL-5 cells with a 200 μL pipette tip. The culture medium was then replaced by fresh complete medium and FRTL-5 cells were allowed to migrate for 24 h or 48 h. Cells migrated for 24 h or 48 h after the wound, were photographed, fixed and then subjected to immunofluorescence experiments. The calculation of the polarity index was performed according to the protocol constructed by Carvalho *et al*,^[29]^ defining the axis of polarity for each cell as the Angle between the edge of the scratch and the axis of cell polarity (nucleus-Golgi vector) (a). The polarity index was calculated according to the following formula:


$$\text{ PolarityIndex }=\sqrt{{\left(\frac{1}{N\sum_{1}^{N}\cos\alpha }\right)}^{2+{\left(\frac{1}{N\sum_{1}^{N}\sin\alpha }\right)}^{2}}}$$

### MRNA stability assay

For PAX8 mRNA stability assay, cell lines stably infected with indicated lentiviruses were treated with the mRNA synthesis inhibitor Actinomycin D (5 μg/mL) for 0 h, 6 h and 12 h. PAX8 mRNA level was detected by RT-qPCR.

### MeRIP-qPCR

Total RNA was extracted from METTL3 knocked-down Nthy cell lines. Each sample containing 5 μg of total RNA was adjusted to 18 μL with RNase-free water, mixed with 2 μL of 10× RNA Fragmentation Buffer, and incubated at 70 °C for 5–6 min to chemically fragment the total RNA into fragments of approximately 200–300 nt in size. The reaction was then terminated by adding EDTA. Subsequently, 30 μL of A/G magnetic beads (#P2108, Beyotime, China) were mixed with 1 μg of anti-m^6^A antibody (#202003, Synaptic Systems, Germany) or normal rabbit IgG (#2729, Cell Signaling Technology, USA) and incubated overnight at 4 °C to allow thorough antibody-bead binding. Next, washed and resuspended antibody-bead complexes were mixed with fragmented RNA and RNase Inhibitor, then incubated at 4 °C for 2–3 h to fully enrich m^6^A-modified RNA fragments onto the magnetic beads. Finally, RNA enriched on the magnetic beads was eluted, reverse transcribed into cDNA, and subjected to RT-qPCR.

### Statistical analysis

All data are expressed as the mean ± SD and the significance of difference of two groups was determined by two-tailed Student’s *t* test. Statistical analysis was performed using SPSS 17.0 software (IBM Corporation, Armonk, NY) and GraphPad Prism 8.0 software (La Jolla, CA, USC). P value < 0.05 was considered statistically significant.

## Results

### Construction of thyrocyte-specific Mettl3 knockout mouse model

**Figure 1:**
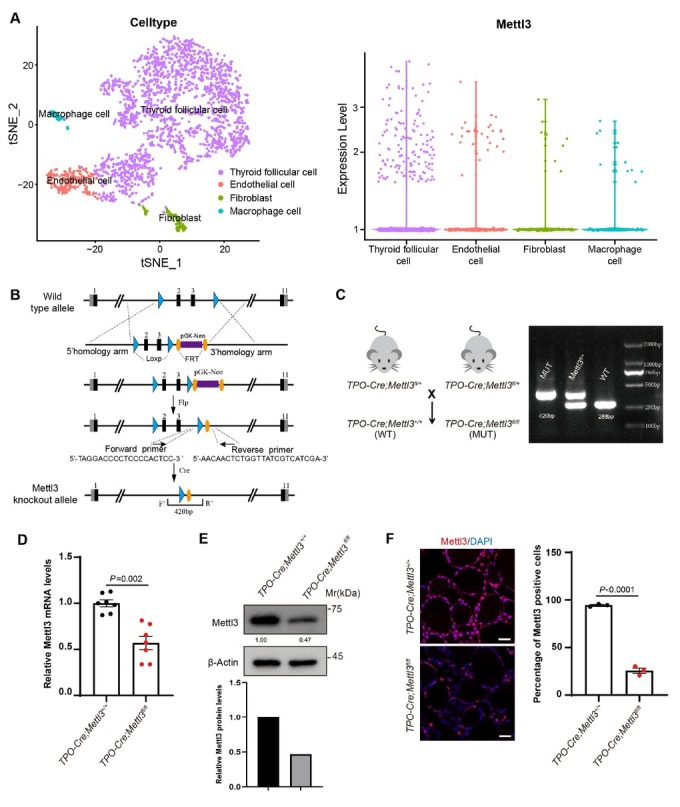
Construction of thyrocyte-specific *Mettl3* knockout mouse model. (A) The expression characteristics analysis of *Mettl3* in thyroid tissue based on single cell sequencing data of mouse thyroid tissue (GSE183963). (B) *Mettl3*^fl/fl^ mice constructed strategy. (C) Cross strategy of thyrocyte-specific *Mettl3* knockout mice model. The genotype of *Mettl3* knockout mice offspring were detected by PCR and the 288bp and 420bp band represent the WT and mutation band respectively. (D–F) qPCR, Western blotting (WB), and Immunofluorescence staining were performed to detect the expression level of *Mettl3* in thyroid tissues (*n* = 3) respectively. DAPI was used to stain the cell nuclei (blue). Scale bar = 20 μm. *N* = 3 biologically independent samples per group and an average of five fields acquired from each sample. Data are shown as the mean ± SD (D, F). *P* values were calculated by unpaired two-tailed Student’s *t* test (D, F).

In order to investigate the involvement of *Mettl3* in thyroid development, we took advantage of accessible mouse thyroid tissue and organoid single cell RNA sequencing data (GSE183963). Significantly, *Mettl3* is abundantly expressed in thyroid follicular cells in comparison to endothelial cells, fibroblast, and macrophage cells within thyroid tissues (Figure 1A). Taken the dominant role of follicular epithelial cells in the endocrine function of thyroid, we constructed *Mettl3* thyroid follicular cell-specific knockout mice by crossing TPO-Cre mice with *Mettl3*^fl/fl^ mice, to have a better view of *Mettl3* functional significance (Figure 1B and 1C). Genetically, wild type (WT) and homozygous mutants presented the 288 bp and 420 bp bands respectively within genotyping assay (Figure 1B and 1C). To check whether *Mettl3* has been knocked out from the thyroid tissues in knockout mice, we detected the mRNA and protein levels of *Mettl3*. The real-time quantitative PCR (qPCR), western blotting (WB), and immunofluorescence (IF) results all showed that the expression level of *Mettl3* in knockout mice (MUT) was significantly reduced, compared to littermate WT mice (Figure 1D–1F). Therefore, the thyroid follicular cell-specific *Mettl3* knockout mice were successfully generated.

### Thyrocyte removal of Mettl3 leads to congenital hypothyroidism-like phenotype

**Figure 2:**
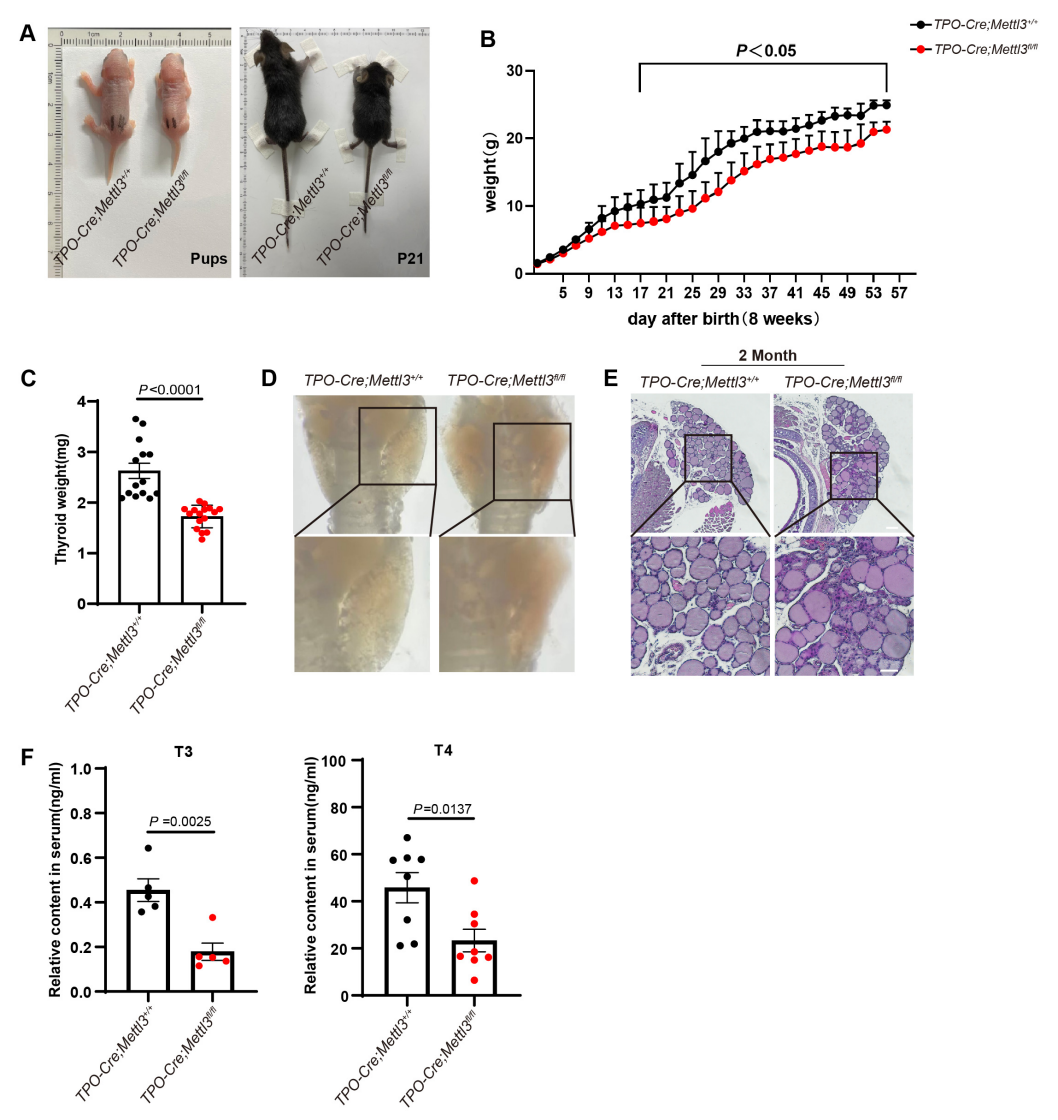
Thyrocyte deficiency of *Mettl3* showed typical CH phenotypes. (A) Body size of TPO-Cre; *Mettl3*^+/+^ (WT) mice and TPO-Cre; *Mettl3*^fl/fl^ (MUT) mice on postnatal day 1 and 21. (B) Body weight of WT and MUT mice (*P* < 0.05, *n* = 6). (C) Thyroid weight of 2-month-old WT mice and MUT mice (*n* = 14). (D) Thyroid tissues morphology of 2-month-old WT mice and MUT mice. (E) H & E staining of 2-month-old WT mice and MUT mice, scale bar = 100 μm. (F) Serum T3 level of 2-month-old WT mice and MUT mice (*n* = 5). (G) Serum T4 level of 2-month-old WT mice and MUT mice (WT, *n* = 7; MUT, *n* = 8). Data are shown as the mean ± SD (B, C, F). *P* values were calculated by unpaired two-tailed Student’s *t* test (B, C, F).

Since Thyroid hormones (TH) are key regulators in essential metabolic processes through normal growth and development, we wonder whether thyrocyte-specific downregulation of *Mettl3* affects physiological progresses. Interestingly, the body sizes and weights of *Mettl3* mutant newborns were greatly reduced in comparison to their WT littermates, the difference tendency escalated more pronouncedly with age growing (Figure 2A and 2B). Anatomically, the weights of *Mettl3* knockout mice thyroid tissue were also significantly reduced (Figure 2C). Further histology analysis showed that thyroid follicles structure of *Mettl3* knockout mice was disorderly arranged, and certain follicles seemed to be replaced by cellular parenchyma (Figure 2D and 1E). Hematoxylin and Eosin staining (HE) also showed that the thyroid follicles of *Mettl3* mutant mice appeared as abnormal fused or enlarged structures (Figure 2E). Normal thyroid follicular formation is the structural basis of thyroid hormone synthesis. We therefore wondered whether the thyroid hormone synthesis was also affected by *Mettl3* knockout. Markedly, the serum triiodothyronine (T3) and thyroxine (T4) levels were significantly lowered in *Mettl3* knockout mice than their littermates WT mice (Figure 2F and 2G). Together, *Mettl3* thyrocyte-specific knockout mice presented typical CH phenotypes, which could be employed as a novel CH mice model for physiological and pathological studies.

### Mettl3 deficiency impaired thyroid follicular cell differentiation

**Figure 3:**
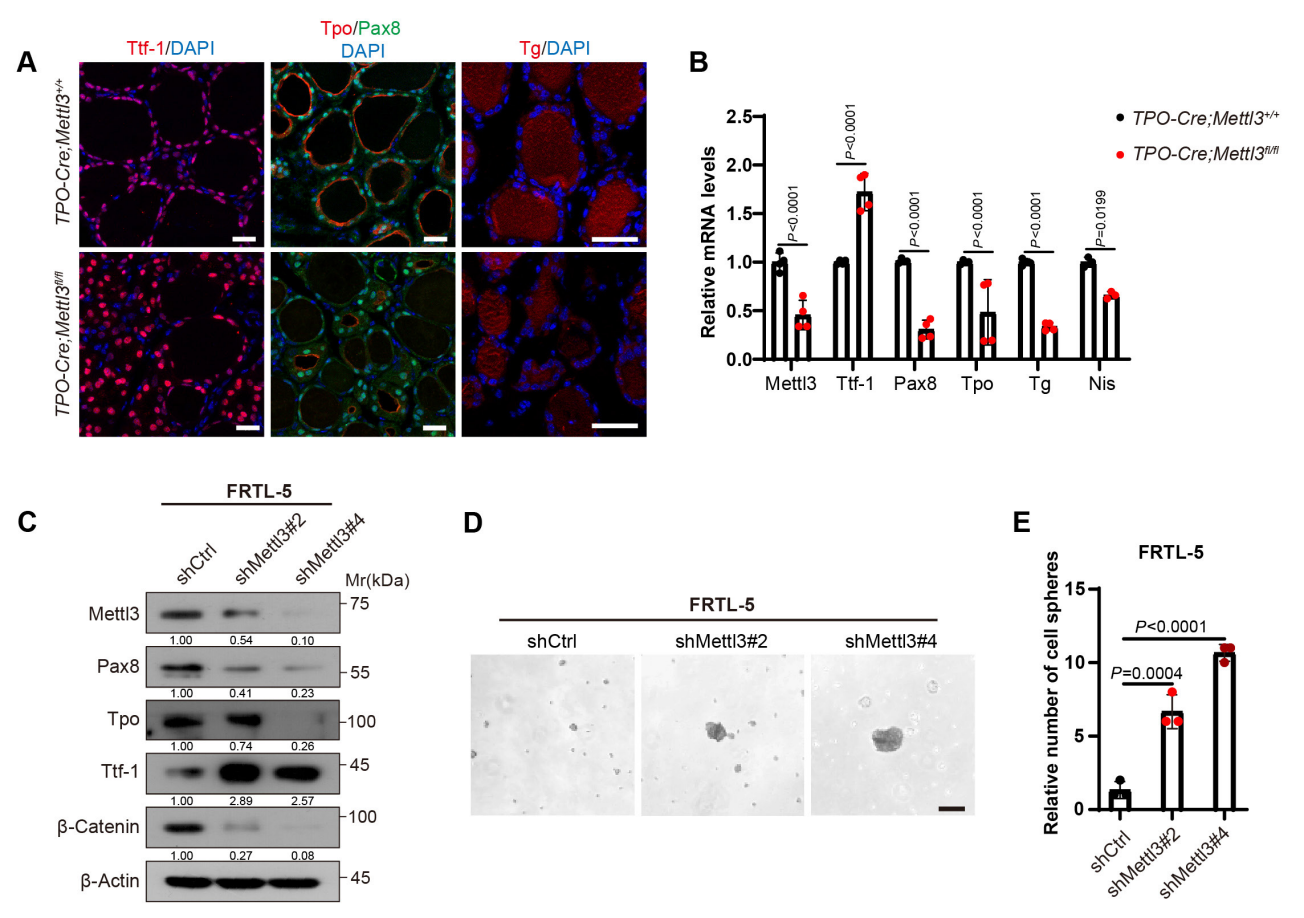
*Mettl3* deficiency impaired thyroid follicular cell differentiation. (A) IF staining of Tpo, Tg and Ttf-1 in 2-month-old WT mice and MUT mice thyroid, scale bar = 20 μm. DAPI was used to stain the cell nuclei (blue). *n* = 3. (B) RT-qPCR assays of Tpo, Tg and Ttf-1 expression level in 2-month-old WT mice and MUT mice thyroid (WT, *n* =3; MUT, *n* = 4). (C) WB analysis of Pax8, Ttf-1, Tg, Tpo, and β-Catenin expression level after knocking out *Mettl3* in FRTL-5 cell lines. (D) Sphere-formation assay after knocking out *Mettl3* in FRTL-5 cell lines. (E) Diagram of quantification of Sphere-formation assay, scale bar = 100 μm. Data are shown as the mean ± SD (B, E). *P* values were calculated by unpaired two-tailed Student’s *t* test (B, E).

The appropriate differentiation progression of follicular cells is critical for thyroid folliculogenesis and TH synthesis. However, In the process of thyroid development, the decrease of differentiation level of thyroid cells is the accumulation and presentation of impaired differentiation level in early development. We therefore asked whether the typical CH phenotype of *Mettl3* knockout mice was partially due to impaired differentiation of thyrocytes. Firstly, expression levels of the critical differentiation markers essential for thyroid hormone synthesis, including TPO, TG, PAX8, and sodium iodide symporter (NIS) were checked. As expected, Tpo, Tg, Pax8, and Nis levels were significantly reduced in Mellt3 knockout thyroids, at both mRNA and protein levels. In comparison, Ttf-1, more stem-fate related thyroid marker, was shifted to the opposite way and showed increasing tendency (Figure 3A and 3B).^[30]^ To further confirm our findings *in vitro*, we constructed knocked-down *Mettl3* in FRTL-5. Accordingly, the expression levels of Tpo and Pax8 were decreased, while that of Ttf-1 was increased (Figure 3C). Functionally, *Mettl3* deficient cells might acquire more progenitor cell-like fate and gave rise to more spheroids in the classical sphere-formation assays (Figure 3D and 3E). These results indicated that *Mettl3* deprivation significantly inhibited thyroid follicular cell differentiation.

### Mettl3 is involved in thyroid follicular polarity patterning

**Figure 4:**
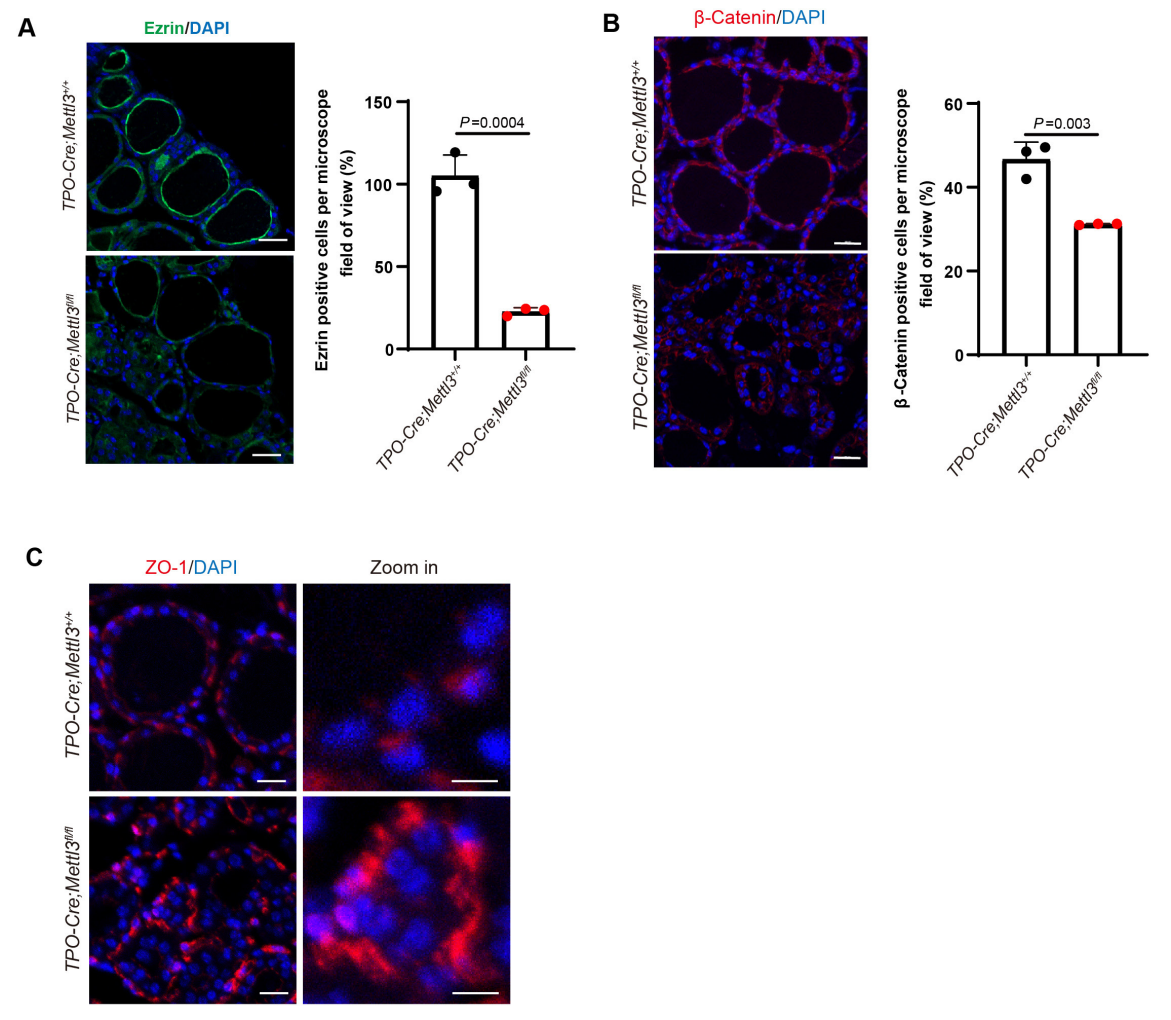
*Mettl3* deficiency attenuated thyroid follicular cell polarity. (A) IF staining of Ezrin in 2-month-old WT mice and MUT mice thyroid, DAPI was used to stain the cell nuclei (blue). *n* = 3, Scale bar = 20 μm. (B) IF staining of β-Catenin in 2-month-old WT mice and MUT mice thyroid, DAPI was used to stain the cell nuclei (blue). *n* = 3, Scale bar = 20 μm. (C) IF staining of ZO-1 in 2-month-old WT mice and MUT mice thyroid, DAPI was used to stain the cell nuclei (blue). *n* = 3, Scale bar = 10 μm. Magnified image scale = 5 μm. Data are shown as the mean ± SD (A, B, C). *P* values were calculated by unpaired two-tailed Student’s *t* test (A, B, C).

Established and ordered thyroid follicular cell polarity is critical for normal folliculogenesis and thyroid function, usually synchronizing with differentiation. The coming question would be how the polarity within those abnormal fused and enlarged thyroid follicles was sustained in *Mettl3* deficient mutants. The Apical-Basel polarization makes thyroid follicular epithelial cells produce special plasma membrane domains that perform different functions, and the epithelial interactions of thyroid follicular cells occur through tight junctions and adhesive junctions, thus ensuring the inner and outer barrier of the thyroid follicles.^[31,32]^ Therefore, tight junction proteins and adhesion proteins often indicate polarity between epithelial cells.^[33, 34, 35]^ By IF staining, Ezrin, the major crosslinker adhesion molecule at the membrane-cytoskeleton interface, was detected on the “apical” side of follicular cells in wild-type follicles, whereas its expression intensity was significantly reduced in *Mettl3* knockdown follicles (Figure 4A). β-Catenin, as an adherens junction protein, colocalizes with cytoskeletal proteins and usually also indicates epithelial polarity,^[36]^ and knockdown of *Mettl3* results in reduced expression (Figure 4B). ZO-1 is a tight junction protein, which is expressed at the tight junction between epithelial cells and belongs to the location-specific junction protein. After *Mettl3* knockdown, the localization of ZO-1 was disordered among follicular cells and it was not expressed in the follicular cell population with abnormal nests, although the overall expression level of ZO-1 did not change significantly (Figure 4C). Collectively, *Mettl3* loss in thyroid tissues may result in destructed thyroid follicular organization and thyroid hormone synthesis, partially through impairing the patterning of polarities.

**Figure 5:**
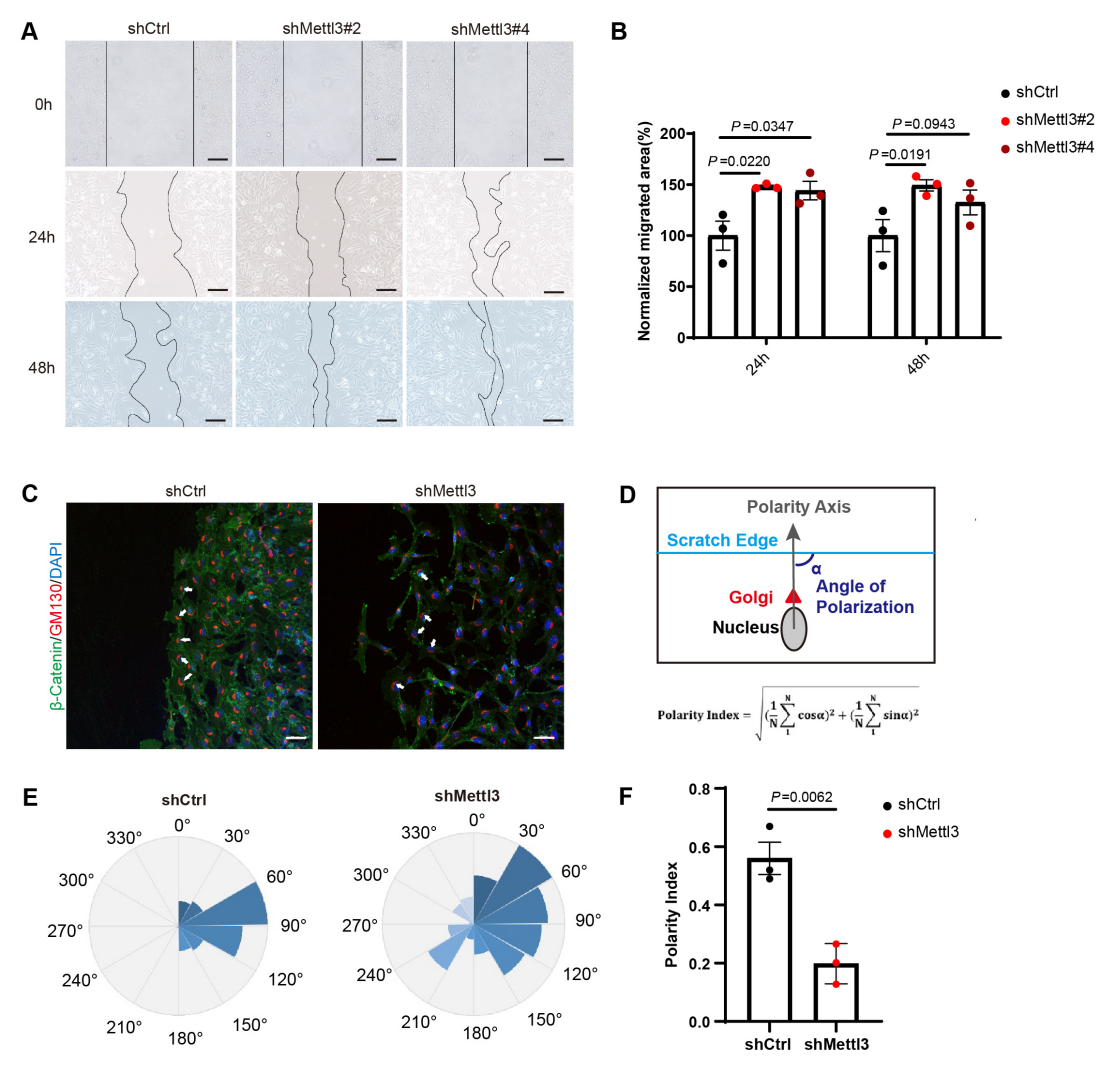
*Mettl3* is essential for maintaining thyrocyte polarity *in vitro*. (A) Scratch-wound assay in *Mettl3* knockdown FRTL-5 cell line, scale bar=50 μm. (B) Quantitative statistics of cell migration area based on (A). (C) IF staining of GM130 and β-Catenin after the FRTL-5 cells were scratched, scale bar = 50 μm. DAPI was used to stain the cell nuclei (blue). (D) Schematic diagram of polarity index calculation. The polarity axis of each cell is defined as the angle (a) between the edge of the scratch and the polar axis of the cell (nucleus-Golgi vector). (E) The angular histogram shows the angular distribution of polar cells. (F) Cell polarity index chart. From 3 independent experiments. Data are shown as the mean ± SD (B, F). *P* values were calculated by unpaired two-tailed Student’s *t* test (B, F).

*In vitro*, we measured the front-rear cell polarity (nucleus-to-Golgi apparatus axis) of *Mettl3* knocked-down FRTL-5 cells at the population level based on the scratch-wound assay in order to further confirm the function of *Mettl3* on thyroid cell polarity. The polarity index (PI), ranging from 1 (strongly polarized) to 0 (random distribution), was applied as the quantification indicator of cell polarity.^[29]^ Interestingly, *Mettl3* knockdown significantly increased the migration capability of FRTL-5 cells (Figure 5A and 5B). Then we wondered whether the increased migration ability was directly related to cell polarity loss, thus calculated the polarity index based on GM130 staining and the polarity formula (Figure 5C–5F). Indeed, the polarity index within *Mettl3* knockdown FRTL-5 was only 0.2, while that of the control group was 0.55 (Figure 5F). Supportively, the expression of cell adhesion molecule β-Catenin was also reduced in *Mettl3* knockdown FRTL-5 cells (Figure 3C). Collectively, knockdown of *Mettl3* in FRTL-5 cell line impaired cell polarity and elevated cell mobility *in vitro*.

### Mettl3 coordinates thyrocyte differentiation and polarity via modulating PAX8

**Figure 6:**
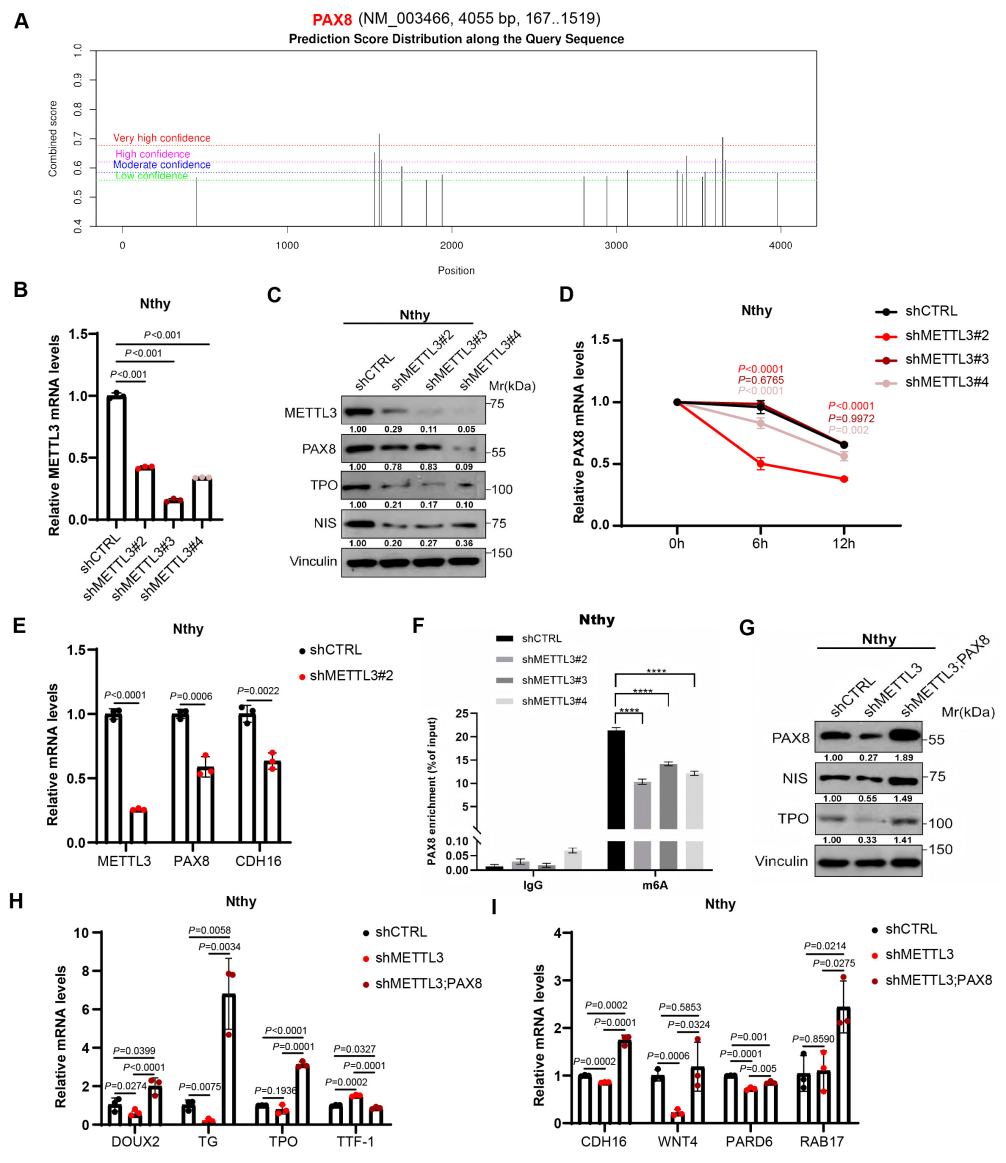
METTL3 regulates thyrocyte differentiation and polarity by modulating PAX8. Potential m^6^A modification sites present in PAX8 mRNA were predicted by the SPRAMP website. (B) METTL3 mRNA expression was detected by RT-qPCR in shMETTL3-transfected Nthy cells. (C) The protein expression levels of METTL3, TPO, PAX8 and NIS were examined by western blotting in shMETTL3-transfected Nthy cells. (D) RT-qPCR analysis of PAX8 mRNA stability of control and METTL3 knockdown Nthy cells. (E) METTL3, PAX8, CDH16 mRNA expression was detected by RT-qPCR in shMETTL3-transfected Nthy cells. (F) m^6^A modification levels of PAX8 mRNA expression was detected by m^6^A-MeRIP-qPCR in shMETTL3-transfected Nthy cells. (G) The protein levels of PAX8, TPO, and NIS were examined by western blotting in Nthy cells which overexpressed PAX8. (H) RT-qPCR assays of DOUX2, TG, TPO and TTF-1 expression levels in Nthy cells which overexpressed PAX8. (I) RT-qPCR assays of CDH16, WNT4, PARD6 and RAB17 expression levels in Nthy cells which overexpressed PAX8. Data are shown as the mean ± SD (B, D, E, F, H, I). *P* values were calculated by unpaired two-tailed Student’s *t* test (B, D, E, F, H, I).

To better understand the potential functional mechanism of METTL3 in thyroid development, we were first curious about the target genes of METTL3 mediated-m^6^A modification. The decreased differentiation characteristics and typical CH phenotype within METTL3 knockout mice drew our attention onto the four thyroid fate determination factors, including PAX8, HHEX, NKX2-1 and FOXE1. Specifically, PAX8 has been also reported to regulate thyrocyte polarity.^[33]^ Moreover, the Online prediction tool (http://www.cuilab.cn/sramp) also located potential m^6^A modification sites within mRNA of PAX8 (Figure 6A). Hence, we wondered whether PAX8 mediates the function of METTL3 in thyroid development. Similar as the alteration within animal model and cell lines, we found that METTL3 knockdown also resulted in down-regulation of PAX8 and other thyroid differentiation-related genes in human thyroid epithelial cells, the Nthy cells (Figure 6B and 6C). Importantly, down-regulation of PAX8 by METTL3 knockdown may be through the mRNA stability modulation which is one of the critical regulatory mechanism of m^6^A modification (Figure 6D). PAX8 has been reported to control apical basal follicle polarization and follicle formation by transcriptionally regulating the expression of the cell adhesion molecule Cadherin-16 (CDH16).^[33]^ Here, we also found that the expression levels of PAX8 and CDH16 (downstream target gene of PAX8) were both decreased upon METTL3 knockdown (Figure 6E), largely due to decreased m^6^A modification levels of PAX8 transcript (Figure 6F). Furthermore, reintroduction of PAX8 into METTL3 deficient Nthy cells significantly reversed the downregulated cell differentiation and impaired cell polarization markers (Figure 6G–I). Therefore, METTL3 may function in maintaining thyrocyte differentiation and polarity by modulating PAX8.

## Discussion

*Mettl3* serves as a pivotal writer gene of m^6^A modification and plays essential roles in various organogenesis and development. In this study, we generated an *Mettl3* thyroid-specific conditional knockout mouse model. The knockout mice exhibited a typical CH phenotype, characterized by reduced body weight, lower T3 and T4 level postnatally. Their thyroid glands appeared abnormal fused and enlarged follicles with compromised polarity status. The expression of genes related to thyroid epithelial cell polarity, differentiation and hormone synthesis were significantly downregulated. We also preliminary demonstrated that the dedifferentiation and loss of polarity in *Mettl3* knockout thyrocytes results from the absence of PAX8 expression. Taken together, our study reveals an important role for METTL3 in thyroid development and highlight the novel regulatory function of the METTL3-PAX8 axis in the initiation and progression of CH.

The lack or an insufficient production of TH at birth is defined as CH. Up to now, the exact molecular mechanism of CH remains obscure, as it is a genetically heterogeneous disease. Suitable CH mouse model may facilitate the discovery of molecular mechanism of CH development.^[6,9]^ Researchers have deepened their understanding of the pathologic morphology of thyroid follicles, cell differentiation and cell polarity status of thyrocytes in CH disease through characterizing knockout mouse models for key thyroid transcription factors, such as TTF-2 or FOXE1, TTF-1 or NKX2–1, PAX8, and HHEX.^[6, 7, 8, 9, 10]^ However, it is reported that less than half of the patients with CH presented mutations in these genes,^[9,15,16]^ suggesting the need to study other genes and/ or mechanisms related to CH.

Our study successfully generated a new CH model, *i.e*., thyrocyte-specific *Mettl3* knockout mouse model. The knockout mice exhibit hypothyroid phenotype upon their born, and the phenotype gets even more pronounced with age progression. Thus, this model not only assists us to understand the essential role of METTL3 in thyroid gland development, but also mimics the development of human CH and provides a reliable tool for discovering the molecular mechanisms of CH.

The majority (80%–85%) of primary CH cases are attributed to developmental defects of the thyroid gland, namely thyroid dysgenesis (TD). Normal thyroid development derived from the differentiation of thyroid precursors or stem cells, and the expression of thyroid hormone synthesis related differentiation genes, such as TPO, TG, NIS, and TSHR, are important markers for thyrocyte differentiation. The thyroid transcription factors FOXE1, TTF-1, PAX8, and HHEX were revealed to direct thyrocyte differentiation and thyroid hormone synthesis related differentiation genes.^[10]^ In this case, mutations or loss of function of these thyroid transcription factors are critical reasons causing TD. Here, we found that knocking out *Mettl3* in thyrocyte significantly inhibited the expression of Pax8 and thyroid hormone synthesis related differentiation genes. On the contrary, the expression level of Ttf-1, as a marker of thyroid precursor or stem cells,^[30]^ was significantly upregulated. With the preliminary experiments which supported the dedifferentiation of thyrocytes being due to PAX8 loss, our results define the mechanism how METTL3 may affect thyrocyte differentiation.

Thyrocytes differentiate from thyroid progenitors that bud from the foregut endoderm as a mass of non-polarized epithelial cells, which reorganize to form prefollicular structures by acquisition of apicobasal polarity. Thus, acquisition of thyrocyte polarity is one of the critical steps for folliculogenesis and thyroid hormone synthesis.^[34,37, 38, 39, 40]^ The thyroid transcription factor PAX8 has been reported to control apical basal follicle polarization and follicle formation by transcriptionally regulating the expression of the cell adhesion molecule CDH16.^[33]^ Our findings indicate the function of PAX8-CDH16 in thyrocyte polarity maintaining is modulated by METTL3 medicated-m^6^A modification, which highlights new perspective in understanding the function of METTL3 in thyroid development.

In conclusion, this study generates a new CH mouse model. METTL3-PAX8 axis appears a novel regulatory mechanism within the initiation and progression of CH. Damage of thyrocyte differentiation and lack of cell polarity could be a critical pathogenesis of CH. Additional validation using human data and expanding clinical value of our findings are important directions for future studies, as the involvement of METTL3 in human CH remains hypothetical and has not yet been clinically validated.

## Supplementary Material

Supplementary Material Details
