# Supplementary material for: Methyltransferase like 3 promotes thyroid folliculogenesis via coordinating cell differentiation and polarization
Source: J Transl Int Med. 2026 Feb 13;14(1):96–107. doi: 10.1515/jtim-2026-0005 (PMC12916267; doi:10.1515/jtim-2026-0005)
Supplement: Supplementary file 1 — Supplementary Material Details [file jtim-2026-0005_sm.pdf]

Supplementary materials

METTL3 promotes thyroid folliculogenesis *via* coordinating cell differentiation and polarization

Ruoyu Jiang[1#], Qibo Zhu[2#], Zhenlei Zhang[3], Xiao He[3], Yifan Liu[3], Ronglin Kan[3], Xianghui He[1], Haixia Guan[2]

Supplementary Table S1: List of shRNA sequences

shCtrl/shCTRL	CCTAAGGTTAAGTCGCCCTCG
shMettl3#2	GCACCCGAAAGATTGAGTTAT
shMettl3#4	GCTGCACTTCAGACGGATTAT
ShMETTL3#2	GCAAGAATTCTGTGACTATGG
ShMETTL3#3	GCTGCACTTCAGACGAATTAT
ShMETTL3#4	GCTCAACATACCCGTACTACA

Supplementary Table S2: List of qPCR primers

**Human gene**	**Strand**	**Sequence**
METTL3	F	CTATCTCCTGGCACTCGCAAGA
METTL3	R	GCTTGAACCGTGCAACCACATC
PAX8	F	ACTACAAACGCCAGAACCC
PAX8	R	AGGGAGGGTTGAATGGTTGC
CDH16	F	AGCCTATCCACCTGGCAGAGAA
CDH16	R	TCTGGTCACGTAGAGGTTTCCC
DOUX2	F	CTGGGTCCATCGGGCAATC
DOUX2	R	GTCGGCGTAATTGGCTGGTA
TG	F	GTTCCTGATTCTGAGTTCCCC
TG	R	GGCAACATTGTCACTTGTCC
TPO	F	AGTCCGTGTCTCTAGCGTC
TPO	R	TGTTGGTCAGGAAGTTTGG
TTF-1	F	CTCGCTCGCTATTTGTTGG
TTF-1	R	ACCAGATCTTGACCTGCGTG
WNT4	F	AGGAGGAGACGTGCGAGAAA
WNT4	R	CGAGTCCATGACTTCCAGGT
PARD6	F	GTCTCAGACCTTGCGATTCTAC
PARD6	R	GGTCCAGAGAGAACCTTCGGA
RAB17	F	ACTTCGGCCAAACTGAACCA
RAB17	R	CCTCCTCGTCGCTTCTCTG
GAPDH	F	CCTGTTCGACAGTCAGCCG
GAPDH	R	CGACCAAATCCGTTGACTCC
**Mouse gene**	**Strand**	**Sequence**
Mettl3	F	CAGTGCTACAGGATGACGGCTT
Mettl3	R	CCGTCCTAATGATGCGCTGCAG
Ttf-1	F	TCCAGCCTATCCCATCTGAACT
Ttf-1	R	CAAGCGCATCTCACGTCTCA
Pax8	F	CAGAAGGCGTTTGTGACAATGA
Pax8	R	TGCACTTTGGTCCGGATGAT
Tpo	F	TGACTTCCAGGAGCACACAG
Tpo	R	GCAAGTTCAGTGATGCCAGA
Tg	F	TGTCCCACCAAGTGTGAAAA
Tg	R	CCAAGGAAAGCTTGTTCAGC
Nis	F	GCTCAGTCTCGCTCAAAACC
Nis	R	CGTGTGACAGGCCACATAAC
β-Actin	F	CCACCATGTACCCAGGCATT
β-Actin	R	CGGACTCATCGTACTCCTGC
